# Dopamine Receptor Activation Reorganizes Neuronal Ensembles during Hippocampal Sharp Waves *In Vitro*

**DOI:** 10.1371/journal.pone.0104438

**Published:** 2014-08-04

**Authors:** Takeyuki Miyawaki, Hiroaki Norimoto, Tomoe Ishikawa, Yusuke Watanabe, Norio Matsuki, Yuji Ikegaya

**Affiliations:** 1 Laboratory of Chemical Pharmacology, Graduate School of Pharmaceutical Sciences, The University of Tokyo, Bunkyo-ku, Tokyo, Japan; 2 Centre for Information and Neural Networks, Suita City, Osaka, Japan; Consejo Superior de Investigaciones Cientificas - Instituto Cajal, Spain

## Abstract

Hippocampal sharp wave (SW)/ripple complexes are thought to contribute to memory consolidation. Previous studies suggest that behavioral rewards facilitate SW occurrence *in vivo*. However, little is known about the precise mechanism underlying this enhancement. Here, we examined the effect of dopaminergic neuromodulation on spontaneously occurring SWs in acute hippocampal slices. Local field potentials were recorded from the CA1 region. A brief (1 min) treatment with dopamine led to a persistent increase in the event frequency and the magnitude of SWs. This effect lasted at least for our recording period of 45 min and did not occur in the presence of a dopamine D_1_/D_5_ receptor antagonist. Functional multineuron calcium imaging revealed that dopamine-induced SW augmentation was associated with an enriched repertoire of the firing patterns in SW events, whereas the overall tendency of individual neurons to participate in SWs and the mean number of cells participating in a single SW were maintained. Therefore, dopaminergic activation is likely to reorganize cell assemblies during SWs.

## Introduction

Hippocampal sharp wave (SW)/ripples are a complex of relatively slow (10–30 Hz) field transients and fast (150–250 Hz) field oscillations and are observed in the hippocampus during slow wave sleep and quiet awake states [1]–[3]. SW/ripples represent transient neuronal synchronization that originates mainly from the CA3 region and propagates to CA1 and the downstream networks [4]. A current hypothesis on learning and memory implies that labile hippocampal memory traces are transferred to a more stable neocortical storage [5] through offline reverberatory replays of behavioral experiences during SW/ripples [6]. In support of this concept, selective suppression of SWs results in an impairment of long-term memory formation [7].

SW/ripples are reported to increase when a rat is rewarded or placed in a novel environment [8], [9]. Because both situations increase dopaminergic neuronal activities [10], [11], dopamine is one of the candidate neuromodulators of the SWs/ripple facilitation, although other modulators such as opioids [12] and orexin [13] are also released during rewarding. Dopaminergic neurons project to various regions in the brain [14], and it remains to be elucidated whether this enhancement is caused directly by dopamine receptor activation in the hippocampus or indirectly by other neural pathways. We hypothesize that hippocampal dopamine directly increases SW/ripple occurrence, based on the following three reasons. First, the ventral tegmental area, a brain reward system, projects its dopaminergic axons to the hippocampus [15]. Second, the extracellular concentration of dopamine in the hippocampus rises in response to novel environmental stimuli [16]. Finally, bilateral injection of a D_1_/D_5_ receptor agonist into the hippocampus enhances memory retention [17], whereas a D_1_/D_5_ receptor antagonist disturbs one-day memory without affecting shorter-term memory [18].

An *in vitro* model of SW/ripples in acute hippocampal slices is usable to examine the effects on pharmacological reagents [19]–[26]. Moreover, the *in vitro* approach using functional multineuron calcium imaging (fMCI) enable us to reveal the spatiotemporal cellular patterns of SW/ripple activity [23], [27]. fMCI is a large-scale optical recording technique that records action potentials *en masse* from a neuron population, by taking advantage of the fact that an action potential reliably evokes a transient increase in the calcium ion concentration of the cell body of the monitored neuron. We exploited this *in vitro* assay system and investigated how dopamine modulates the occurrence of SWs and their internal structures. Our data showed that transient dopamine receptor activation induced a persistent increase in SW occurrence via dopamine D_1_/D_5_ receptors. This facilitation was accompanied by a reorganization of spiking patterns in SWs.

## Results

**Figure 1:**
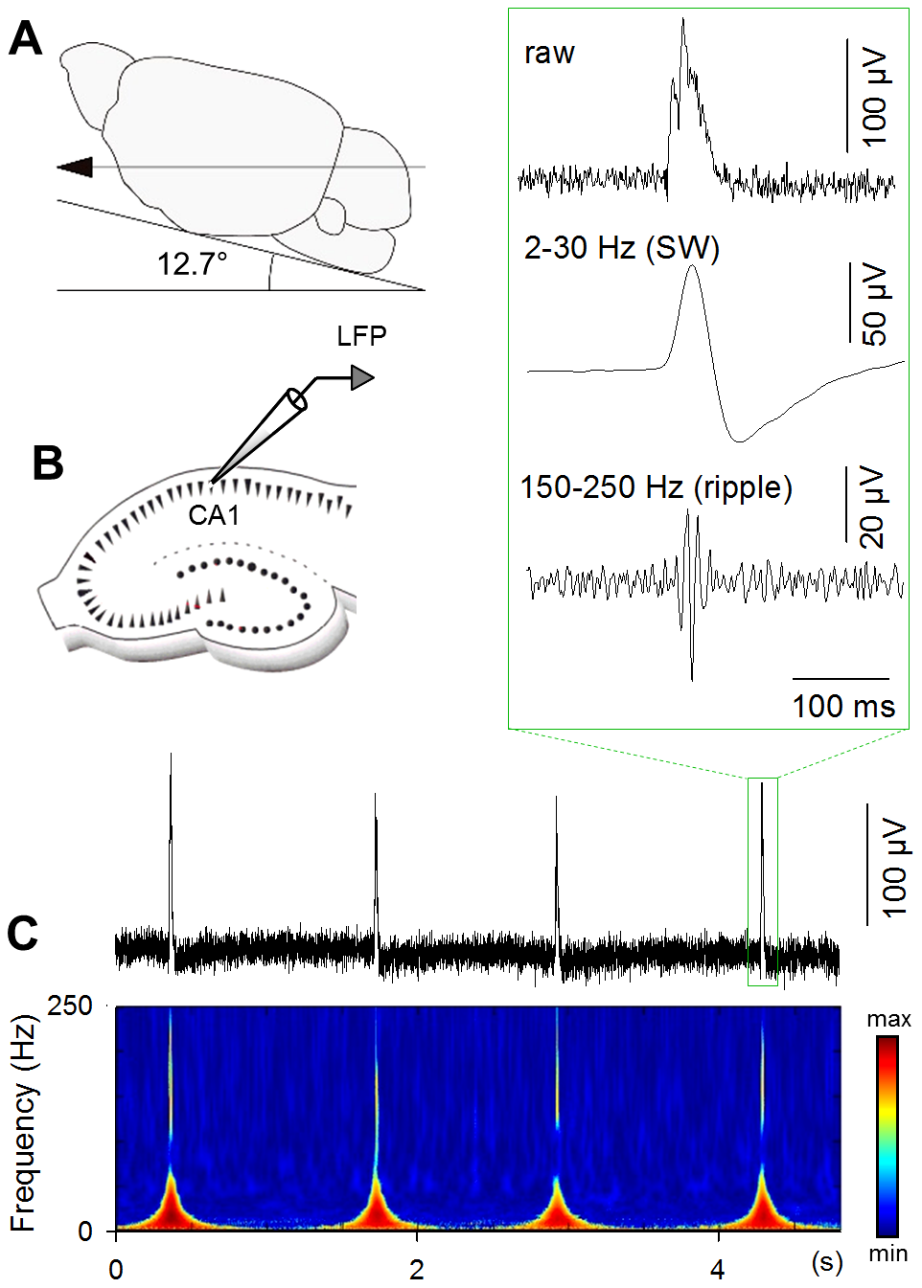
SW/ripples occur spontaneously in obliquely sliced hippocampal preparations. **A.** The brains of 3-to-4-week-old mice were sliced at an angle of 12.7° in the fronto-occipital direction. **B.** LFPs were recorded from CA1 stratum pyramidale of the hippocampal slices. **C.** An example of LFP recording. The top raw trace was wavelet-analyzed into the bottom power spectrogram. The inset indicates magnifications of raw (top), 2–30-Hz-filtered (middle, SW), and 150–250-Hz-filtered (bottom, ripple) traces.

Local field potentials (LFPs) were recorded from CA1 stratum pyramidale of hippocampal slices that were obliquely cut at an angle of 12.7° in the fronto-occipital direction (Fig. 1A and 1B). Slices that were perfused with artificial cerebrospinal fluid (aCSF) at 7–9 ml/min for > 1.5 h spontaneously exhibited SWs, as reported previously [23]. The SW events occurred together with ripple oscillations (Fig. 1C). The mean frequency of SW events was 0.42±0.17 Hz (mean ± SD of 55 slices) and did not differ from that reported in *in vivo* studies [28].

**Figure 2:**
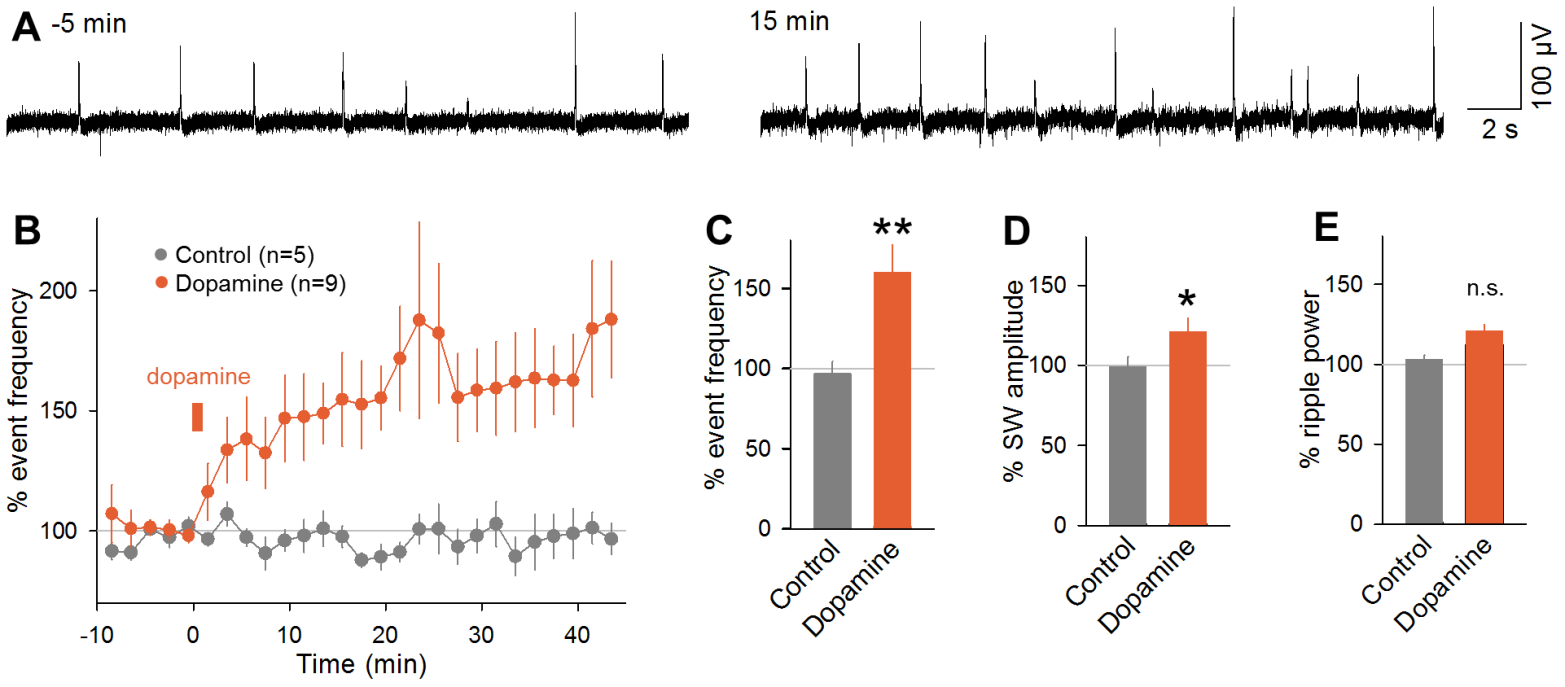
Bath application of dopamine induces long-lasting facilitation of SW events. **A.** Representative traces of CA1 LFPs recorded 5(left) and 15 min after (right) 1-min application of 1 µM dopamine. **B.** Time course of the percentage of the frequency of SW events relative to the mean value during a 6-min period prior to the dopamine application. Slices were bath-perfused with 1 µM dopamine for 1 min at time 0–1 min. Control slices were perfused continuously with normal extracellular solution. **C–E.** The mean ratios of the SW event frequency (C), the SW amplitude (D), and the ripple power (150–250 Hz) (E) were calculated from the −5-to-0 min period and the 30-to-35 min period. **P = 0.0082, *t*_8_ = 3.49; *P = 0.045, *t*_8_ = 2.37, paired *t*-test *versus* the −5-to-0 min period. Data are the means ± SEMs of 9 and 5 slices from 7 and 3 mice.

To examine the effect of dopamine receptor activation on SWs, we perfused hippocampal slices with 1 µM dopamine for 1 min. Immediately after dopamine washout, SW events started to increase gradually in frequency. Within 10–20 min, the effect reached a steady state at 160.0±17.2% relative to the baseline level and persisted at least for our observation period of 45 min (Fig. 2A–C, mean ± SEM of 9 slices, *P* = 0.0082, *t*_8_ = 3.49, paired *t*-test *versus* the baseline period). The bath application of dopamine also increased the peak amplitude of the LFP deflections during SWs to 120.6±8.7% (Fig. 2D, *P* = 0.045, *t*_8_ = 2.37, paired *t*-test). This result suggests that after the dopamine challenge, CA1 networks received stronger synaptic inputs from CA3 networks during SWs. On the other hand, the mean power of ripple oscillations, which were measured between 150 Hz and 250 Hz in the fast Fourier transform of LFP traces, did not significantly change (Fig. 2E, *P* = 0.069, *t*_8_ = 1.65, paired *t*-test), suggesting that dopamine exerted little effect on the population firing level of CA1 neurons; however note that the tendency of the ripple power change was in the same direction as the SW amplitude change and could become significant if the number of data was increased.

**Figure 3:**
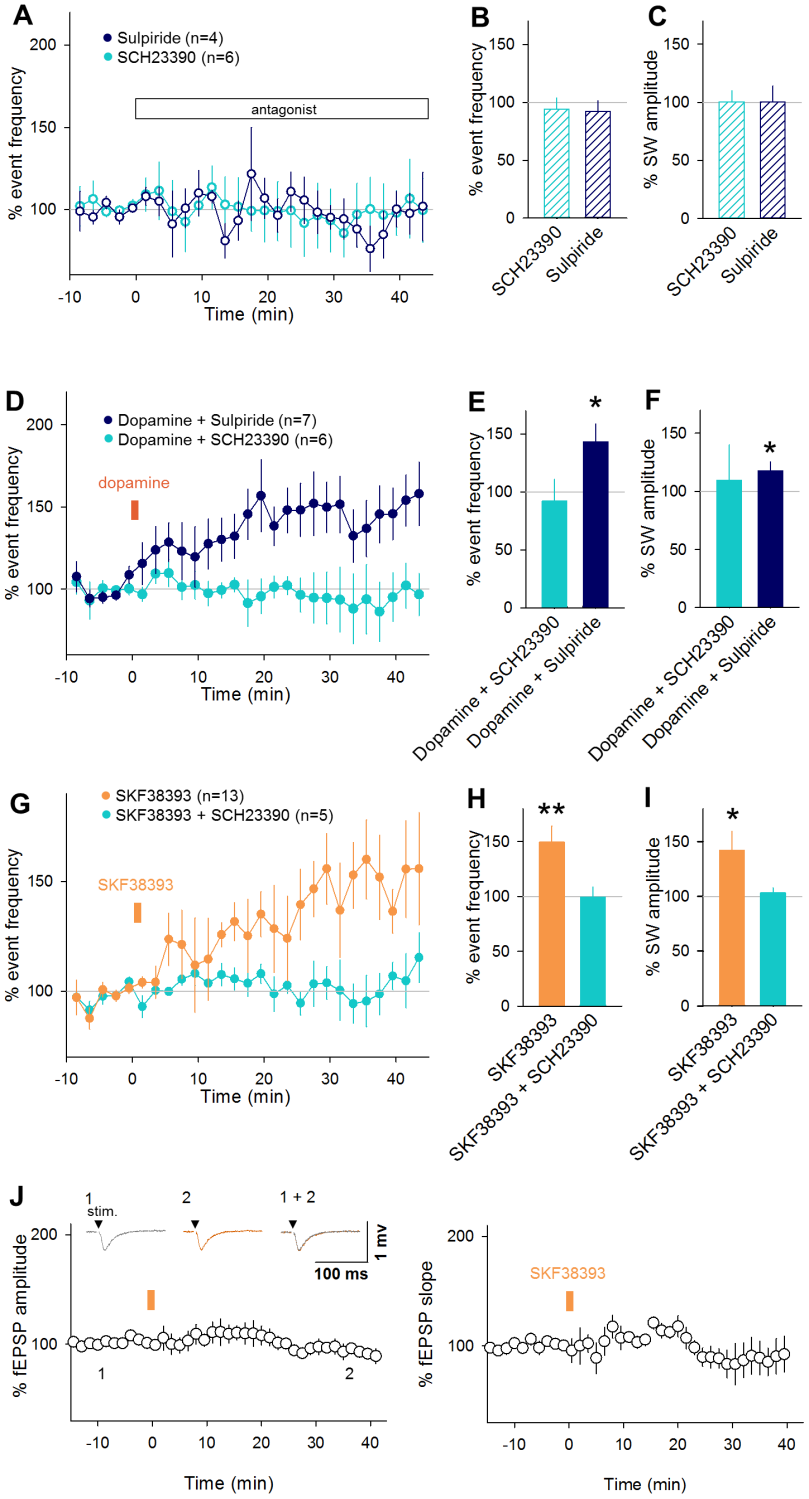
D_1_/D_5_ receptor activation is responsible for dopamine induced SW facilitation. **A.** Time course of the percentage of the frequency of SW events relative to the mean value during the pre-application period while the slices were perfused with 0.1 µM SCH23390, a D_1_/D_5_ receptor antagonist, and 1 µM sulpiride, a D_2_ receptor antagonist, from time 0–45 min. **B–C.** The mean SW event frequency (B) and the mean SW amplitude (C) at time 30–35 min. **D.** Slices were perfused with 1 µM dopamine for 1 min at time 0 in the continuous presence of 0.1 µM SCH23390 or 1 µM sulpiride. **E–F.** The mean SW event frequency (E) and the mean SW amplitude (F) at time 30–35 min. *P = 0.032, *t*_6_ = 2.78, *P = 0.035, *t*_6_ = 2.71, paired *t*-test *versus* the −5-to-0 min period. Data are the means ± SEMs of 7 and 6 slices from 4 and 3 mice, respectively. **G.** Slices were perfused with 30 µM SKF38393 at time 0 in the absence (orange) and the continuous presence (light blue) of 0.1 µM SCH23390, a D_1_/D_5_ receptor antagonist. **H–I.** The mean SW event frequency (H) and the mean SW amplitude (I) at time 30–35 min. **P = 0.0017, *t*_12_ = 4.02 (H); *P = 0.017, *t*_12_ = 2.78 (I), paired *t*-test *versus* the −5-to-0 min period. Data are the means ± SEMs of 13 and 5 slices from 10 and 3 mice, respectively. **J.** While fEPSPs evoked by field stimulation of Schaffer collaterals were recorded from CA1 stratum radiatum, slices were perfused with 30 µM SKF38393 for 1 min at time 0. Time changes in fEPSP amplitudes (left) and slopes (right) are plotted as mean ± SEMs of 3 slices from 3 mice. The insets indicate example traces at two time points.

To examine the subtypes of dopamine receptors involved in this phenomenon, we applied dopamine in aCSF containing either 0.1 µM SCH23390, a dopamine D_1_/D_5_ receptor antagonist, or 1 µM sulpiride, a dopamine D_2_ receptor antagonist. Either antagonist alone did not affect SW frequency or amplitude during the baseline period before dopamine application (Fig. 3A–C; frequency: sulpiride, *P* = 0.45, *t*_3_ = 0.86, SCH23390, *P* = 0.74, *t*_5_ = 0.35; amplitude: sulpiride, *P* = 0.97, *t*_3_ = 0.045, SCH23390, *P* = 0.96, *t*_5_ = 0.057, paired *t*-test). In the presence of SCH23390, dopamine did not increase the frequency or amplitude of SWs (Fig. 3D and 3E, 92.1±18.7%, *P* = 0.69, *t*_5_ = 0.42; Fig. 3F, 109.4±30.7%, *P* = 0.77, *t*_5_ = 0.31; paired *t*-test), whereas it did in the presence of sulpiride (Fig. 3D and 3E, 143.1±15.5%, *P* = 0.032, *t*_6_ = 2.78, paired *t*-test). The same tendency was observed in their amplitudes (Fig. 3F, 117.8±7.56%, *P* = 0.035, *t*_6_ = 2.71, paired *t*-test). Thus, D_1_/D_5_ receptors are likely to mediate dopamine-induced SW facilitation. To further confirm this, we applied 30 µM SKF38393, a D_1_/D_5_ receptor agonist, for 1 min. SKF38393 induced a long-lasting facilitation of the SW event frequency (Fig. 3G and 3H, 149.6±14.6%, *P* = 0.0017, *t*_12_ = 4.02, paired *t*-test). This facilitation exhibited a time course similar to the effect of dopamine (Fig. 2) and was also accompanied by an increase in the SW amplitude (Fig. 3I, 142.0±17.3%, *P* = 0.017, *t*_12_ = 2.78, paired *t*-test). The effect of SKF38393 was not observed in the presence of 0.1 µM SCH23390 (Fig. 3H and 3I, *P* = 0.96, *t*_4_ = 0.055; Fig. 3I, *P* = 0.47, *t*_4_ = 0.79, paired *t*-test). In the following experiments, therefore, we used SKF38393, but not dopamine, to stimulate D1/D5 receptors specifically.

Tetanic stimulation to Schaffer collaterals is known to induce long-term potentiation (LTP) at CA3-CA1 synapses and CA3 recurrent synapses, and also to increase the incidence of SW/ripples [21], which suggests that synaptic strengthening can facilitate SW occurrence. In addition, D_1_/D_5_ receptor activation is known to induce LTP-like facilitation at CA3-CA1 synapses, although the effect appears slowly [29]. In our experimental system, we confirmed that 1-min treatment with 30 µM SKF38393 did not induce LTP at CA3-CA1 synapses within 45 min during which period SWs were already facilitated (Fig. 3J; amplitude, *P* = 0.56, *t*_2_ = 0.69; slope, *P* = 0.66, *t*_2_ = 0.52, paired *t*-test). Thus, LTP is unlikely to account for the SW facilitation observed in this study.

During SW/ripples, specific subsets of neurons fire action potentials [21], [24], [30]–[33], exhibiting a rich repertoire of the internal patterns of SWs (*i.e.*, various combinations of activated neurons) [24], [34], [35]; however, LFP recordings alone cannot determine whether the dopamine-increased occurrence of SWs reflected an increase in the repetitions of the same SW patterns or an increase in the SW pattern variability. To address this question, we used fMCI, an optical technique that records spikes through action potential-evoked calcium elevations in the cell bodies of the focused neurons [36], because unit activities were not evident in our LFP recordings. A drawback of the fMCI technique is its low time resolution due to slow calcium signal kinetics, so that we could not resolve individual spikes during a SW/ripple event. In the present study, therefore, we focused on the combinations (patterns) of neurons that participated in individual SW events, rather than the temporal spike sequences during the SWs.

**Figure 4:**
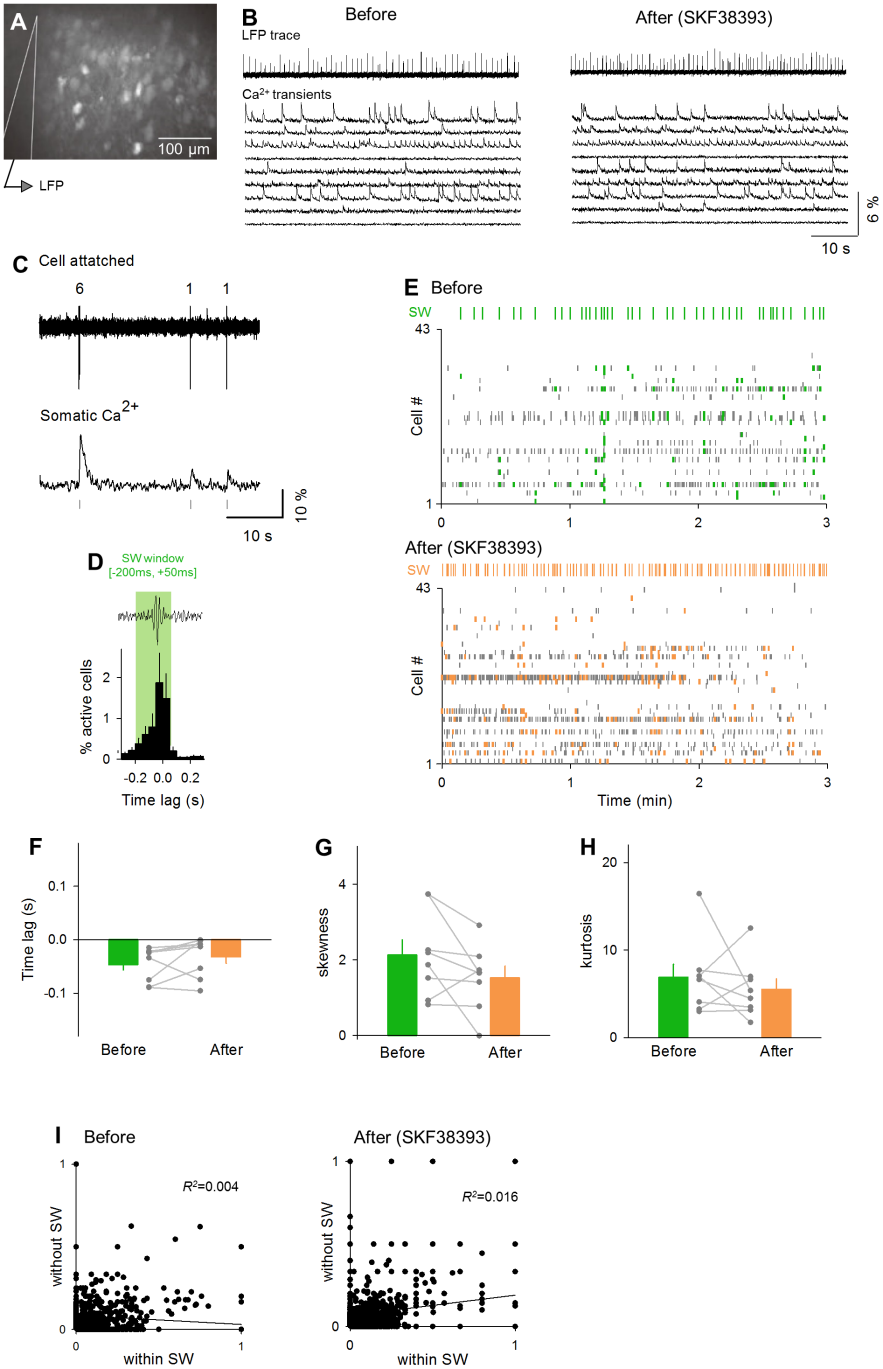
Neuronal activities during SWs are optically imaged. **A.** A confocal image of the CA1 stratum pyramidale in an OGB1-loaded slice. The location of an LFP electrode is shown by the white lines. **B.** Example of calcium transients from 9 cells and LFP trace, before (left) and after (right) SKF38393 application. **C.** Simultaneous cell-attached recording and calcium imaging. Numbers of spikes are represented above each spike. Spike timimgs detected from the calcium trace are shown by bars below the trace. **D.** A peri-SW time histogram of calcium events. Data are the means ± SEMs from 8 slices, including a total of 7,709 calcium events emitted by 199 cells. The time period between −200 ms and 50 ms relative to the SW peak was defined as a SW window (shadow). Calcium events during this time window are regarded as SW-locked activities. **E.** Representative raster plots of calcium events 0–3 min before (top) and 15–18 min after (bottom) the bath application of 30 µM SKF38393. Green or orange bars above each raster plot indicate the time stamps of individual SW events. Thick dots in the rastergram indicate SW-locked activities. **F–H.** Comparisons of peri-SW time histograms from each slices in 3 parameters; Time lag of the histogram peak from the SW peak (F), skewness of the histograms (G) and kurtosis of the histograms (H). **I.** Comparisons of the co-activation probability of any given pairs of cells that participated at least once in SW before (left) and after (right) the drug application. Each dot indicates a single neuron pair.

Oregon Green BAPTA1 AM (OGB1) was bolus-injected into CA1 stratum pyramidale (Fig. 4A), and the fluorescence of OGB1 was monitored at 50 frames per second from CA1 cells in an area of approximately 400×250 µm^2^, which contained an average of 45.8±15.4 OGB1-loaded neurons (mean ± SD of 8 slices). Neurons spontaneously exhibited transient Δ*F*/*F* rises in their cell bodies (Fig. 4B). The calcium transients were associated with spikes of the neurons, and even single spikes were detectable in Δ*F*/*F* traces (Fig. 4C). LFPs were simultaneously recorded from CA1 stratum pyramidale in the same microscopic field (Fig. 4A and 4B).

We plotted a peri-SW time histogram for the timings of calcium events relative to the SW peak time (Fig. 4D, *n* = 7,709 calcium events in 252 cells from 8 slices). The histogram exhibited an evident peak at time 0 ms. The mean firing rates started to increase at approximately −200 ms and suddenly dropped to nearly zero at 50 ms. Therefore, we defined SW-locked activities as calcium events that occurred within a time window between −200 ms and 50 ms relative to the SW peak time. Not all calcium events were time-locked to SWs; 252 neurons (68.9%) of a total of 366 neurons in 8 slices exhibited at least one calcium activity during our imaging period of 3 min. Among these active neurons, 199 neurons (79.0%) were SW participants; they exhibited at least one SW-locked activity. Of a total of 7,709 calcium transients emitted by all 252 active cells, 3,597 (46%) were SW-locked activities. On average, a single SW event recruited 4.0±5.6% of the total OGB1-loaded 366 neurons (mean ± SD of  = 1,871 SWs). A single SW-participating neuron participated in 4.8±4.9% SW events (mean ± SD, *n* = 199 neurons).

To examine how these patterned SW activities are modified by D_1_/D_5_ dopamine receptor activation, we bath-applied 30 µM SKF38393 for 1 min. The imaging period was limited to a total of 6 min to minimize laser illumination-induced photobleaching and phototoxicity. We compared the activity patterns between two 3-min periods immediately before and about 20 min after application of SKF38393 (Fig. 4E). For each period, we analyzed 795 and 1,076 SW events, which involved 3,714 and 3,995 calcium transients, respectively. To quantify the effect of D_1_/D_5_ receptor activation on the distribution of SW-associated firing (Fig. 4D), we calculated the mean time lag from the SW peaks (Fig. 4F) and the skewness (Fig. 4G) and kurtosis (Fig. 4H) of the peri-SW time histogram of calcium transients. None of these parameters differed significantly between the periods before and after SKF38393 application, suggesting that D_1_/D_5_ receptor activation does not change the SW-locked firing properties (mean, *P* = 0.35, *t*_7_ = 1.01; skewness, *P* = 0.12, *t*_7_ = 1.77; kurtosis, *P* = 0.48, *t*_7_ = 0.75, paired *t*-test).

We also analyzed the spike rates during SWs and during the remaining periods between SWs. The mean rates of calcium transients during SWs were 1.5±2.1 and 1.6±1.9 activities/min/cell before and after SKF38393 application, respectively (mean ± SD of 366 cells, *P* = 0.55, *t*_7_ = 0.62, paired *t*-test), whereas the mean activity rates during SW-free periods were 1.7±1.2 and 1.9±2.0 activities/min/cell before and after SKF38393 application (*P* = 0.66, *t*_7_ = 0.46, paired *t*-test). We also compared the level of spike synchronization between a pair of neurons. We defined *N*_1&2_/(*N*_1_+*N*_2_−*N*_1&2_) as a synchrony index, where *N*_1_ and *N*_2_ denote the total numbers of calcium transients in neuron #1 and neuron #2, respectively, and *N*_1&2_ denotes the total number of calcium transients emitted jointly by these neurons in a time window of 250 ms. The synchrony index did not correlate between the ‘in-SW’ and the ‘inter-SW’ periods (Fig. 4I left; *R* ^2^ = 0.004), suggesting that SWs actively recruit different cell assemblies from neuron populations synchronizing during SW-free periods. This tendency was maintained after SKF38393 application (Fig. 4I right; *R* ^2^ = 0.016).

**Figure 5:**
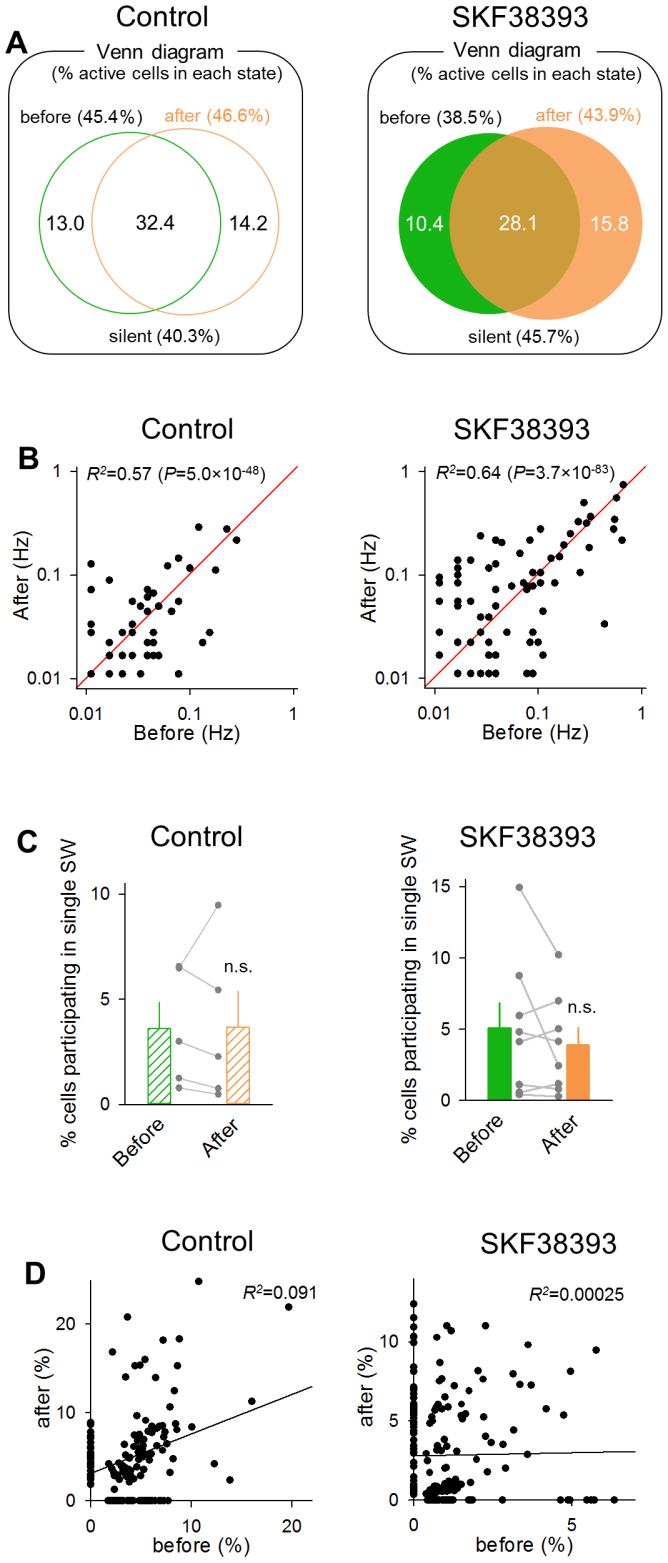
SKF38393 preserves SWs-participating neurons. **A.** A Venn diagram of the population of cells that participated in at least one SW event during the 3-min periods before and after the application of control aCSF (left) or 30 µM SKF38393 (right). The values indicate the percentage of cells involved in the corresponding states to the total cells. The two populations overlapped significantly. Control: *P* = 1.85×10^−5^ *versus* the chance level (21.2%), *Z* = 4.28; SKF38393: *P* = 1.62×10^−8^ versus the chance level (16.9%), *Z* = 5.65, Z-test for a proportion. **B.** Relationship of the frequencies of SW-locked activities of individual neurons before and after control aCSF (left) or 30 µM SKF38393 (right) administration. Each dot indicates a single neuron. Control: *R* ^2^ = 0.57, *P* = 5.0×10^−48^, *t*_251_ = 18.3; SKF38393: *R* ^2^ = 0.64, *P* = 3.7×10^−83^, *t*_364_ = 25.5, *t*-test of a correlation coefficient. **C.** The mean percentage of cells that participated in a single SW event to the total cells before and after aCSF or SKF38393 administration. Each gray dataset represents a single slice. Control: *P* = 0.93, *t*_4_ = 0.098; SKF38393: *P* = 0.25, *t*_7_ = 1.23, paired *t*-test. Data are the means ± SEMs of 5 or 8 slices. **D.** Comparison of the mean amplitudes of SW-locked calcium transients from 150 and 199 SW participants before and after the drug application. Control: *R* ^2^ = 0.091, SKF38393, *R* ^2^ = 0.00025, *P* = 0.13, *Z* = 1.53, *Z*-test for two correlation coefficients. Each dot indicates a single neuron.

Next, we examined the population dynamics. During the baseline period, 38.5% neurons were SW participants (Fig. 5A right, *n* = 141 cells). This ratio slightly increased to 43.9% (*n* = 161 cells) after SKF38393 application, but this change was not statistically significant (*n* = 366 cells; *P* = 0.41, *Z* = 1.48, *Z*-test for the equality of two proportions). The SW participants before and after SKF38393 application shared 28.1% common neurons. This overlap ratio was significantly higher than the stochastically expected ratio of 16.9% (*P* = 1.62×10^−8^, *Z* = 5.65, *Z*-test for a proportion), suggesting that dopamine receptor activation did not actively change SW-participating cell populations. To confirm this idea, we have examined how activity of neuronal populations drift spontaneously under the control conditions without drug application [37]. We continuously perfused aCSF without SKF38393 (control aCSF) and compared the activity patterns between two 3-min periods intervened by 20 min, which corresponded to the time course of the experiments with SKF38393 application. For the two periods, we analyzed 377 and 405 SW events, which involved 1,465 and 1,321 calcium transients, respectively (*n* = 253 cells in 5 slices from 5 mice). SW participants were 45.4% (115 cells) in the first 3-min period and 46.6% (118 cells) in the second 3-min period, and 32.4% (82 cells) participated in both periods (Fig. 5A left). The overlap ratio was again significantly higher than the chance ratio of 21.2% (*P* = 1.85×10^−5^, *Z* = 4.28, *Z*-test for a proportion). Importantly, the overlap ratios did not differ between control and SKF38393 experiments (*P* = 0.30, *Z* = 1.15, *Z*-test for the equality of two proportion), supporting our idea that SKF38393 did not actively change SW-participating cell populations.

At a more microscopic level, the SW-locked activity frequencies of individual neurons were positively correlated before and after SKF38393 application (Fig. 5B right, *R* ^2^ = 0.64, *P* = 3.7×10^−83^, *t*_364_ = 25.5, *t*-test for a correlation coefficient). A similar tendency was observed in control slices without SKF38393 application (Fig. 5B left, *R* ^2^ = 0.57, *P* = 5.0×10^−48^, *t*_251_ = 18.3), and the correlation coefficients did not differ between control and SKF38393 experiments (*P* = 0.57, *Z* = 0.57, *Z*-test for two correlation coefficients). Thus, dopamine receptor activation did not alter the tendency of SW participation of individual neurons. Moreover, dopamine receptor activation did not change the mean number of neurons participating in single SW events (Fig. 5C right; *P* = 0.25, *t*_7_ = 1.23, paired *t*-test), as did in the control group (Fig. 5C left, *P* = 0.93, *t*_4_ = 0.098, paired *t*-test). The mean amplitudes of calcium transients in each cell were little correlated before and after SKF38393 application, as were in the control group (Fig. 5D, control: *R* ^2^ = 0.091, SKF38393, *R* ^2^ = 0.00025, *P* = 0.13, *Z* = 1.53, *Z*-test for two correlation coefficients).

**Figure 6:**
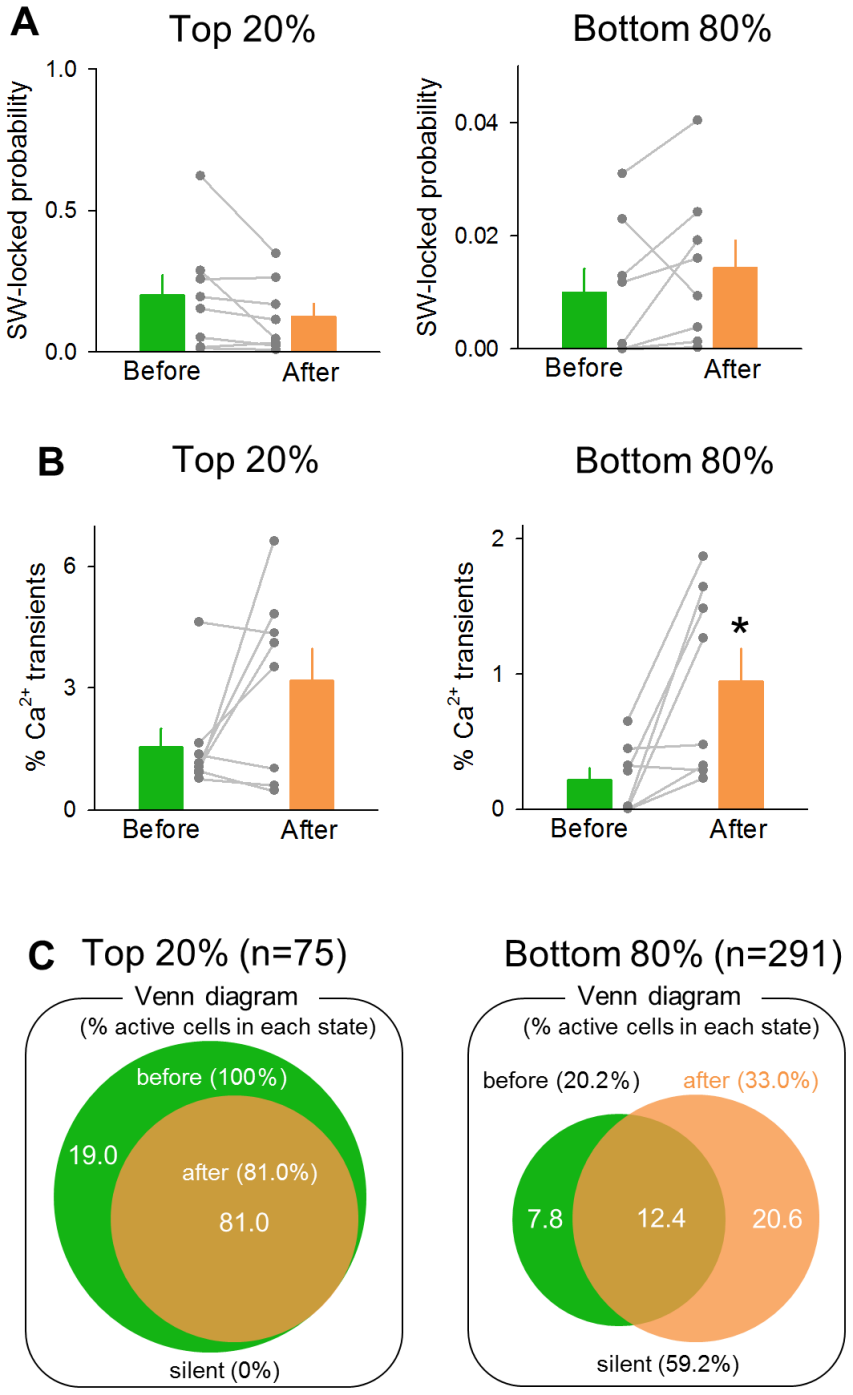
Putative pyramidal neurons are more affected by SKF38393 than putative interneurons. **A.** The mean SW-locked probability of cells with the top 20% SW-locked probability (left) and that of the bottom 80% SW-locked probability (right). **B.** The mean amplitude of calcium transients of the top 20% SW-locking cells (left) and that of the bottom 80% cells (right; *P = 0.016, *t*_7_ = 3.14, paired *t*-test). **C.** A Venn diagram of the population of cells that participated in at least one SW event during the 3-min periods before and after the application 30 µM SKF38393 for the top 20% SW-locking cells (left) and the bottom 80% cells (right).

We sought to examine whether the effect of D_1_/D_5_ receptor activation was different between excitatory and inhibitory neurons. In our imaging experiments, the neuron types were not separable by the OGB1 appearance or the firing rates [38], but it is known that interneurons constitute 20% of the total CA1 cells [39] and that interneurons are more tightly time-locked to SW events compared to excitatory neurons [40]. Therefore, we expediently regarded the top 20% cells for the ‘SW-locked’ probability, the ratio of SW-locked activities to the total SW events, during the baseline period as putative interneurons and the others as putative pyramidal neurons. For both groups, we compared the mean SW-locked probability, the mean amplitude of calcium transients, and the population of SW-participating cells before and after SKF38393 application (Fig. 6). For the SW-locked probability, either groups did not show statistically significant changes (top 20%, Fig. 6A left; *P* = 0.11, *t*_7_ = 1.82, paired *t*-test, bottom 80%, Fig. 6A right; *P* = 0.23, *t*_7_ = 1.32, paired *t*-test). However, SKF38393 significantly increased the amplitudes of SW-locked calcium transients of putative pyramidal neurons, but not in putative interneurons (top 20%, Fig. 6B left; *P* = 0.09, *t*_7_ = 1.98, paired *t*-test, bottom 80%, Fig. 6B right; *P* = 0.016, *t*_7_ = 3.14, paired *t*-test). Putative interneurons did not exhibit a cell population shift after SKF38393 application, and the populations of SW participants were more overlapped before and after SKF38393 application, compare to those in control slices (Fig. 6C; *P*<10^−20^, *Z* = 12.0, *Z*-test for the equality of two proportion). Therefore, D_1_/D_5_ receptor activation seemed to exert a more effect on pyramidal neurons than on interneurons.

**Figure 7:**
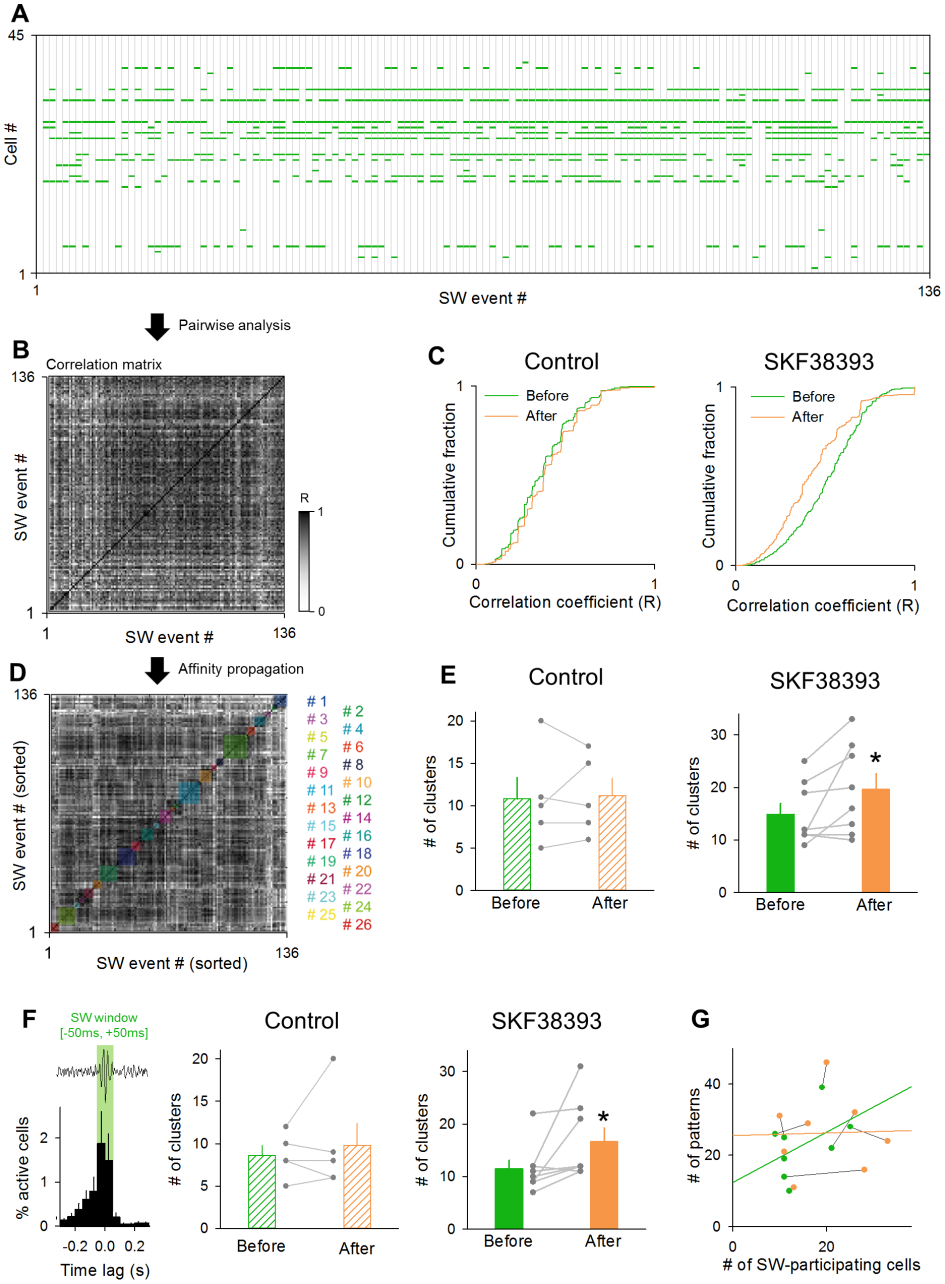
SKF38393 increases the repertoires of SW-relevant firing patterns. **A.** Representative spatiotemporal patterns of SW-locked activities in 136 SW events during the observation period of 3 min. **B.** The correlation coefficients between patterns of SW-participating neurons were calculated for all possible SW pairs. The representative correlation matrix was obtained from 136 SWs shown in A. **C.** Cumulative distribution of the correlation coefficients before (green) and after (orange) perfusion with control aCSF [left, 795 SW events (before) and 1,076 (after) from 8 slices] and SKF38393 [right, 795 SW events (before) and 1,076 (after) from 8 slices]. **D.** The affinity propagation algorithm separated 136 SW events in B. into 26 SW subgroups, indicated by different colors. **E.** The mean number of the SW subgroups before and after the application of control aCSF (left) and SKF38393 (right). Each gray dataset indicates a single slice. *P = 0.030, *t*_7_ = 2.25, paired *t*-test. Data are the means ± SEMs of 5 or 8 slices. **F.** The same analysis as E was repeated a time window between −50 and +50 ms relative to the SW peak (left). The mean numbers of the SW subgroups before and after the application of control aCSF (left) and SKF38393 (right) are shown in the bar graph. *P = 0.036, *t*_7_ = 2.12, paired *t*-test. Data are the means ± SEMs of 8 slices. **G.** The relationship between the number of SW-participating neurons and of SW patterns. Data obtained from the same slice are connected with black line. Before (green); *R* ^2^ = 0.21, *P* = 0.24. After (orange); *R* ^2^ = 8.3×10^−4^, *P* = 0.95.

We then focused on the internal patterns of SW events. First, we extracted SW-locked activities and generated a SW-activity matrix in which SW-locked activities were arranged in a space of the cell number (row) *versus* the SW event number (column) (Fig. 7A). We binarized this matrix so that the matrix elements with SW events were ‘1’ (activated), whereas the others were ‘0’ (not-activated). To evaluate the pattern similarity between two given SWs, we calculated the correlation coefficient between two binary vectors of the corresponding SW events. We repeated this calculation for all possible SW pairs in the SW-activity matrix and created a correlation matrix (Fig. 7B). As a whole, the correlation coefficients were lower after SKF38393 application compared to those observed before the application (Fig. 7C right; *P* = 2.7×10^−15^, *D* = 0.184, Kolmogorov-Smirnov test), whereas the distribution of the correlation coefficients did not shift in the control group (Fig. 7C left). Thus, SWs became more dissimilar to one another after dopamine D_1_/D_5_ receptor activation. We thus hypothesized that SKF38393 increases a repertoire of SW patterns. To examine this possibility, we applied the affinity propagation algorithm, an iterative clustering method [41], to the correlation matrix. Based on the SW pattern similarity, this algorithm unsupervisedly determined the total number of SW subgroups (clusters) that existed in the matrices (Fig. 7D) [42]. We found that the number of SW clusters increased after SKF38393 application (Fig. 7E right; *P* = 0.030, *t*_7_ = 2.25, paired *t*-test, *n* = 8 slices), but not after no drug application (Fig. 7E left; *P* = 0.78, *t*_4_ = 0.30, paired *t*-test, *n* = 5 slices). We repeated the same analysis for a time window between −50 and +50 ms relative to the SW peak, which period is more often used in previous literature about SWs. We obtained the same result (Fig. 7F; control: *P* = 0.54, *t*_4_ = 0.68, paired *t*-test, *n* = 5 slices; SKF38393: *P* = 0.036, *t*_7_ = 2.12, paired *t*-test, *n* = 8 slices). Thus, we conclude that dopamine D_1_/D_5_ receptor activation led to the emergence of different patterns of SWs. To exclude the possibility that the increased SW patterns were merely due to an increase in the statistical chance of cell combinations, which could came from a slight increase in the number of SW-participating neurons (see Fig. 5A right), we examined the relationship between the number of SW-participating neurons and the number of patterns. These two values were not correlated (Fig. 7G, Before (green); *R* ^2^ = 0.21, *P* = 0.24. After (orange); *R* ^2^ = 8.3×10^−4^, *P* = 0.95). Thus, the effect of the increased chance of possible cell combinations on the increased SW patterns was, if any, minimal.

**Figure 8:**
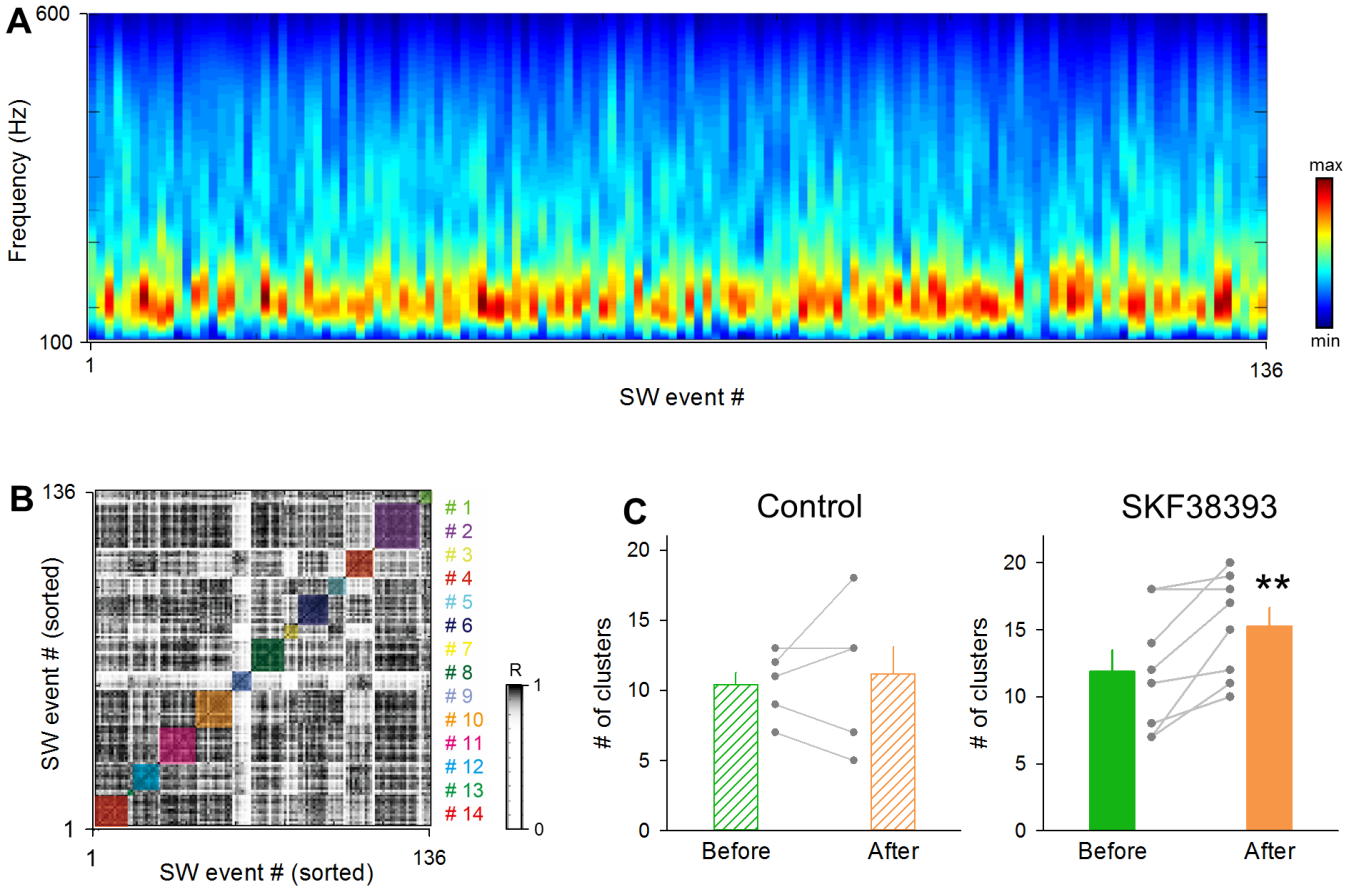
SKF38393 increases the repertoires of SW-relevant LFP spectra patterns. **A.** Representative fast Fourier transform spectra of 136 SW events during the pre-treatment baseline period of 3 min. **B.** The correlation matrix obtained from the 136 SWs was sorted by the affinity propagation and was clustered into 14 SW subgroups. **C.** The mean numbers of the SW subgroups before and after administration with control aCSF (left) and SKF38393 (right). Each gray dataset indicates a single slice. **P = 0.0059, *t*_7_ = 3.38, paired *t*-test. Data are the means ± SEMs of 8 slices.

Finally, we sought to confirm this conclusion at a different level of neuronal activity. The SW patterns are reported to be reflected in SW-related LFP spectrum patterns [30], [31], [43], [44]. Indeed, we found diverse SW patterns in the LFP spectra (Fig. 8A). We thus applied the same clustering method to the correlation coefficients between the LFP spectrum patterns (Fig. 8B). The number of SW clusters increased after SKF38393 treatment (Fig. 8C right; *P* = 0.0059, *t*_7_ = 3.38, paired *t*-test, *n* = 8 slices), while this tendency is not observed in the control group (Fig. 8C left; *P* = 0.69, *t*_4_ = 0.53, paired *t*-test, *n* = 5 slices).

## Discussion

We demonstrated that a brief bath-application of dopamine to hippocampal slices induced a long-lasting increase in the SW event frequency through dopamine D_1_/D_5_ receptor activation. The D_1_/D_5_ receptor activation did not largely change SW-participating neurons and the number of neurons involved in single SWs, but it reorganized combinations of neurons co-participating in SWs and thereby expanded the diversity of the internal structures of SWs. D_1_/D_5_ receptor activation is reported to suppress cholinergically induced gamma oscillations [45]. On the other hand, a decreased level of cholinergic tone is associated with the SW/ripple-rich state of hippocampal networks [46], and we have previously demonstrated that cholinergic receptor activation suppresses the occurrence of SW/ripples [23]. Recent studies have demonstrated that SW/ripple-related firing increases in frequency in slices that received cholinergic stimulation, and such specific enrichment of SW/ripples may be linked to the efficiency of memory consolidation [47], [48]. Therefore, dopamine-induced increases in SW repertoires may be associated with an enrichment of episodic-like contexts encoded in memories.

CA3 neurons that fire in ripple events are known to increase in number when rats are rewarded [9]. In our study, dopamine receptor activation did not significantly change the power of CA1 ripple oscillations or the number of CA1 neurons that were activated in single SW events, but it readily increased the amplitude of SWs. SWs are recorded as sharp sources of the field potentials in CA1 stratum pyramidale and are likely to reflect synaptic inputs from CA3 neurons [34] (although caution must be taken to interpret what the SW components mean, especially when LFPs were recorded from the soma; because they do not directly measure the pure sum of synaptic activity (or spiking activity) of individual excitatory neurons and are often contaminated with active sources of somatic inhibition). This idea that dopamine-induced increase in the SW size suggests an increased level of synaptic inputs is consistent with the fact that after reward stimulation, ripple events come to recruit more CA3 neurons [9]. fMCI from the CA3 region is required to confirm this hypothesis, but unfortunately, it is extremely difficult to load CA3 neurons with calcium indicators, due to unknown reasons (unpublished data).

An apparent contradiction is that neither the ripple power nor the number of calcium events in a SW event accompanied the increased SW amplitude, suggesting that stronger synaptic inputs did not lead to more spike outputs. During SW/ripples, pyramidal cells as well as inhibitory interneurons, such as basket cells and bistratified cells, increase their firing rates [35]. We have recently shown that the SW amplitude correlates with the strength of SW-relevant phasic inhibitory inputs as well as excitatory inputs [40]. Therefore, synaptic inputs to CA1 pyramidal cells are likely balanced between excitation and inhibition during SWs [24], [49], [50] and thereby may provide the homeostatic properties of CA1 network excitability, as opposed to increased CA3 excitation during SW/ripples.

We directly evaluated the pattern change in individual SWs by utilizing the affinity propagation algorithm. This algorithm allowed us to detect the latent cliqueness underlying SWs based on their internal patterns. Interestingly, although dopamine receptor activation did not change the overall tendency of individual neurons to participate in SWs or the mean number of neurons involved in a single SW, it readily reorganized subsets of neurons that participated jointly in SWs. As a result, dopamine increased the total number of SW categories. However, possible is another trivial explanation that the increased repertoires was merely a mathematical consequence of an increase in the total number of active neurons. In other words, the effect may be due partly to more visibility of the latent network through an emergence of previously silent interactions. Although the slight increase in SW-participated neurons after SKF38393 administration was not statistically significant, our results cannot alone determine whether higher activation of individual neurons led to an apparent increase in neuronal ensemble repertoires or whether the network actively recruited a more number of neuronal interactions in order to increase the repertoires.

We imaged calcium activities at 50 Hz and cannot dissolve sequential activation of SW-participating neurons [51]–[54]. It will be interesting to see dopamine-induced changes in spike sequence patterns. To this end, faster image acquisition is needed. Moreover, fMCI using chemical dyes does not allow us to distinguish pyramidal neurons from GABAergic interneurons. Our data would be more informative if the activity patterns can be dissociated between different types of neurons. Using a targeted patch-clamp recording technique, we have recently demonstrated that interneurons fire action potentials during almost all SW events [40]. Because their firing patterns are rather homogenous compared to a rich repertoire of pyramidal neuron activity. Therefore, we believe that interneurons are unlikely to finely modulate (or be modulated by) the SW activity patterns.

Under *in vivo* conditions, sets of place cells that were activated prior to reward stimuli are preferentially reactivated in subsequent SW/ripples. Our *in vitro* work failed to show that the increased repertories of SWs arose from increased occurrence of a specific set of “rewarded” SWs that had rarely emerged during the baseline period. To assess whether dopamine facilitates SWs in a pattern-specific manner, investigations using online closed loop-driven, SW pattern-triggered activation of dopamine receptors is necessary. Recently available dopamine-related optogenetic tools [55] or caged-dopamine reagents [56] will help this approach.

In conclusion, this study provides an insight into the cellular mechanisms for reward-enhanced memory consolidation in terms of SW/ripples. It is also intriguing to interpret our data in relation to the dopamine hypothesis of schizophrenia. Dopamine release is increased in patients with schizophrenia [57], and an experimental mouse model of schizophrenia exhibits an aberrantly increased level of SW/ripples [54]. Combining a pattern-specific closed-loop system with behavioral assessments will reveal a causal link between dopaminergic SW modulation and its behavioral consequence.

## Methods

### Animal ethics

Experiments were performed with the approval of the animal experiment ethics committee at the University of Tokyo (approval number: P24-8) and according to the University of Tokyo guidelines for the care and use of laboratory animals.

### Drug

Dopamine dihydrochloride (Sigma-Aldrich, St Louis, MO), SCH23390 (Tocris-Bioscience, Bristol, UK), (S)-(–)-sulpiride (Sigma-Aldrich), and SKF38393 hydrochloride (Sigma-Aldrich) were dissolved at 10 mM in water and stocked at 4°C. Immediately before use, they were diluted to the final concentration with aCSF containing (in mM): 127 NaCl (Nacalai Tesque, Kyoto, Japan), 1.6 KCl (Wako, Tokyo, Japan), 1.24 KH_2_PO_4_ (Nacalai Tesque), 1.3 MgSO_4_ (Nacalai Tesque), 2.4 CaCl_2_ (Wako), 26 NaHCO_3_ (Wako), and 10 d-glucose (Wako).

### Slice Preparation

Acute slices were prepared from the hippocampal formation of male 3-to-4-week-old ICR mice. Mice were anesthetized with ether and decapitated, and a posterior brain block (400 µm thick) was obliquely cut at an angle of 12.7° in the fronto-occipital direction using a vibratome in ice-cold oxygenated cutting solution consisting of (in mM) 222.1 sucrose, 27 NaHCO_3_, 1.4 NaH_2_PO_4_, 2.5 KCl, 1 CaCl_2_, 7 MgSO_4_, 0.5 ascorbic acid [23], [58]. Slices were transferred to an interface chamber containing oxygenated aCSF at 35°C and were allowed to recover for at least 1.5 h. On average, 2–4 slices were obtained from one brain.

### Electrophysiological recording

Experiments were performed in a submerged chamber perfused at 7–9 ml/min with oxygenated aCSF at 35–37°C. LFPs were recorded from CA1 stratum pyramidale using borosilicate glass pipettes (1–2 MΩ) filled with aCSF. Signals were amplified by MultiClamp 700B (Molecular Devices, Union City, CA, USA), digitized at 10,000 Hz and filtered with a band of 1–2,000 Hz by pCLAMP 10 (Molecular Devices). Offline analysis was conducted using custom-made MATLAB routines (MathWorks, Natick, MA, USA). To detect SW/ripples, LFP traces were band-pass filtered at 2–30 Hz and were thresholded at 4 times above the SD of the baseline noise. The detected SW events were scrutinized by eye and manually rejected if they were erroneously detected. Cell-attached patch-clamp recordings were obtained from CA1 pyramidal neurons with an Axopatch 700B amplifier (Molecular Devices). Borosilicate glass pipettes (4–7 MΩ) were filled with aCSF. Signals were low-pass filtered at 1–2 kHz, digitized at 20 kHz and analysed with pCLAMP 10.2 software (Molecular Devices). fEPSPs were recorded at room temperature. Stimuli were delivered through bipolar tungsten electrodes placed in the CA1 stratum radiatum to stimulate Schaffer collaterals, and fEPSPs were recorded from the CA1 stratum radiatum using glass pipettes filled with aCSF. Test pulses with a duration of 100 µs was given every 30 s, and the stimulus intensity was set at about 30% of the maximum amplitude of fEPSPs. Changes in fEPSPs were expressed as a percentage change in their amplitudes and maximal slopes, relative to the mean values during the baseline period of −15 to 0 min).

### Optical recording

fMCI was conducted using acute slices loaded locally with OGB1-AM [36]. Fluorophores were excited at 488 nm and visualized using a 507-nm long-pass emission filter. Videos were at 50 frames/s using a 16× objective (CFI75LWD16xW, Nikon, Tokyo, Japan), a Nipkow-disk confocal microscope (CSU-X1; Yokogawa Electric, Tokyo, Japan), and a cooled EM-CCD camera (iXon DU897, Andor, Belfast, UK). The fluorescence change was measured as (*F_t_*−*F_0_*)/*F_0_*, where the *F_t_* is the fluorescence intensity at a given time point; *F_0_* is the baseline. Spike-elicited calcium transients were automatically detected and visually inspected [59]. Our automatic algorithm was composed of two steps, *i.e.*, a conditioning (learning) phase and a test (spike detection) phase. In the conditioning phase, the parameters were tuned to give the most appropriate probability of spike detection from sample datasets. In the test phase, the algorithm with the best tuned parameters was used to extract spike signals from datasets different from those used in the conditioning phase. The data used for conditioning was collected from 10 cells. All routines were written in MATLAB (Math Works). Co-activation probability within a SW was calculated for any pair of cells that participated at least once in SWs. The probability for cell A and cell B was given as *N*(A∩B)/(*N*(A)+*N*(B)−*N*(A∩B)), where *N*(A∩B) is the number of SWs in which cell A and cell B fired in the same SW events, and *N*(A) (or *N*(B)) is the number of SWs in which cell A (or cell B) participated. The co-activation probability without SWs was calculated for any pair of cells that fired at least once during the period without SWs *i.e.* activities that occurred outside the SW period *i.e.*, from −200 ms to +50 ms relative to the SW peak. Two spikes that occurred within 250 ms was defined as co-activated firing. The co-activation probability for cell A and cell B was *N*(A∩B)/(*N*(A)+*N*(B)−*N*(A∩B)), where *N*(A∩B) is the total number of co-activated events, and *N*(A) (or *N*(B)) was the total number of spikes of cell A (or B) during SW-free periods. A time bin of 20 ms was used to evaluate the shapes of the distribution of calcium transients that occurred within 500 ms relative to the SW peak. For each neuron, the total number of calcium transients within SW periods was divided by the recording period (3 min) and was defined as the SW-locked activity frequency of the neurons.

### Statistical Analysis

The affinity propagation was performed with custom-written MATLAB software [41]. The correlation matrix, which represents the similarity between all possible SW pairs, was calculated from a Cell-SW matrix and was treated with the algorithm. The clustering was conducted by iteratively passing “messages” between the data points until it reached the equilibrium. Specifically, for the first step, the “responsibility” was passed from a point to any other points. The responsibility means how comparatively suitable a point is to be the exemplar for another point rather than any other point. For the second step, the “availability” was passed from a point to any other points. The availability means how comparatively appropriate it would be for any other points to pick message-sending point as its exemplar. The affinity propagation for LFP traces was performed in the same way, but now using a spectrum-SW matrix in which each row corresponded with the ripple power in fast Fourier transform at a band filter of 100–600 Hz. The bin used was 1 Hz. We reported data as the means ± SEMs unless specified otherwise. Student's *t*-test, *Z*-test for the equality of two proportions, *Z*-test for a proportion, *t*-test for a correlation coefficient, *Z*-test for two correlation coefficients, and Kolmogorov-Smirnov test were used to assess the significance of the differences. *P*<0.05 was considered statistically significant.
